# The effect of laparoscopy on mast cell degranulation and mesothelium thickness in rats

**DOI:** 10.1186/s12893-020-00775-y

**Published:** 2020-05-24

**Authors:** Hery Poerwosusanta, Gunadi, Zairin Noor, Ika Kustiyah Oktaviyanti, Karyono Mintaroem, Bambang Pardjianto, Moch Aris Widodo, Edi Widjajanto

**Affiliations:** 1 Doctoral Study Program, Faculty of Medicine, Universitas Brawijaya, Malang, Indonesia; 2 Department of Surgery, Ulin General Hospital, Faculty of Medicine, Universitas Lambung Mangkurat, Banjarmasin, Indonesia; 3 Pediatric Surgery Division, Department of Surgery, Faculty of Medicine, Public Health and Nursing, Universitas Gajah Mada /Dr. Sardjito Hospital, Yogyakarta, Indonesia; 4 Department of Surgery, Ulin General Hospital, Faculty of Medicine, Universitas Lambung Mangkurat, Banjarmasin, Indonesia; 5 Department of Anatomical Pathology, Faculty of Medicine, Universitas Lambung Mangkurat, Banjarmasin, Indonesia; 6 Department of Biomedical Science, Faculty of Medicine, Universitas Brawijaya, Malang, Indonesia; 7 Department of Biomedical Science, Faculty of Medicine, Universitas Brawijaya, Malang, Indonesia; 8 Department of Biomedical Science, Faculty of Medicine, Universitas Brawijaya, Malang, Indonesia; 9 Department of Biomedical Science, Faculty of Medicine, Universitas Brawijaya, Malang, Indonesia

**Keywords:** Laparoscopic procedure, mast cell infiltration and degranulation, mesothelium thickness, oxidative stress, *Rattus norvegicus*

## Abstract

**Background:**

Laparoscopy induces adhesion due to ischemia-reperfusion injury. However, the detail pathomechanism is poorly understood. This study aimed to investigate the impact of laparoscopy on mast cell and mesothelium morphological changes in the rat.

**Methods:**

Forty-nine males of Sprague-Dawley *Rattus norvegicus* were divided into four groups: a) control and b) intervention groups P1, P2, and P3 that underwent 60 min laparoscopic using carbon dioxide (CO_2_) insufflation at 8, 10, and 12 mmHg groups, respectively. Serum hydrogen peroxide (H_2_O_2_), catalase (CAT), superoxide dismutase (SOD), malondialdehyde (MDA), and oxidative stress index (OSI) levels were determined 24 h after laparoscopy. Histopathological analyses of mast cell infiltration and degranulation and mesothelium thickness in the liver, greater omentum, mesenterium, small intestine, and peritoneum were performed 7 days after the procedure.

**Results:**

H_2_O_2_, MDA, and OSI levels were significantly increased in the intervention groups compared with the control (*p*<0.05), while the SOD and CAT levels were decreased in the intervention groups compared with the control (*p*<0.05). Mast cell infiltration and degranulation were higher in the intervention groups than in control (*p*<0.05), while the mesothelium thickness was significantly lower in the laparoscopic groups than in control (*p*<0.05). Interestingly, the decrease in mesothelium thickness was strongly associated with the increase in mast cell infiltration and degranulation (*p*<0.01).

**Conclusions:**

Our study shows that laparoscopy in rats increases mast cell infiltration and degranulation, which also results in and correlates with a decrease in mesothelial thickness.

## Background

At present, laparoscopy is the preferred and standard surgical technique. This procedure requires insufflation of carbon dioxide (CO_2_) for better visualization but affects the mesothelium membrane [1] and changes the local morphology to a certain degree [2]. Although laparoscopy decreases the risk of mesothelial damage up to 25% compared to laparotomy [3], detachment of mesothelial cells during laparoscopic surgery still occurs and causes adhesion formation [4]. Information about pathomechanism and how to prevent mesothelial cell detachment is rarely found.

Mast cells are specific cells that mature in peripheral tissues, including the peritoneum [5], and can produce proteases (e.g., tryptase, chymase, and carboxypeptidase A3), which are retained in cytosolic granules during the resting phase. Mast cells can be activated through several pathways and degranulation results in the release of proteases in a protective response to injuries. Effect of tryptase promotes extracellular matrix deposition (ECM) by activating proteinase-activated receptors (PAR)-2 in ECM fibroblasts. Chymase affects tight junction proteins and causes cell release from the basement membrane [6]. Mast cell degranulation thought to play a role in mesothelial cell damage. Damage to mesothelial cells through degranulation of mast cells can cause complications, that is, intra-abdominal adhesion.

Laparoscopy results in ischemia-reperfusion injury, causing a transient hypoxia of abdominal organs [2]. These effects will decrease after desufflation [2]. During ischemia, cells in the peritoneum develop reactive oxygen species (ROS), which can damage tissues and cause adhesions [7]. Ten percent of the immune cells commonly found in the mesothelium layer are mast cells [8]. Pneumoperitoneum induces free radicals production by mesothelium and also mast cells [9]. Our study investigated the impact of laparoscopy on mast cells and mesothelium morphological changes in rats. In this study, we used rats because they have similar physiological and immunological aspects with human [10, 11].

## Methods

### Animals

According to the Federer formula [12], 49 males (200–250 g and 20–25 weeks) Sprague-Dawley *Rattus norvegicus* from the Abadi Jaya farm in Yogyakarta, Indonesia, were used for this study. This study followed the main principles of experimental animals, i.e., 3R (replacement, reduction, and refinement) and 5F (freedom of hunger and thirst, freedom from discomfort, freedom of pain, injury, or disease, freedom to fear and distress, and freedom to express natural behaviour). Male rats were preferred because they are stronger, and have higher blood volume than the females, and to avoid the estrus hormonal phase in the female rats [13] The rats were treated according to standard conditions for single housing and breeding. Heart rate, body temperature, and activity were checked for 7 days of acclimation. The room was maintained in a controlled 12 h light/dark cycle at 20 ± 2°C temperature and relative humidity of 45–70% [14]. The rats were randomized into one control group, and three intervention groups: the control group (n=7) did not undergo pneumoperitoneum, while the intervention groups P1 (n=14), P2 (n=14), and P3 (n=14) were given 8, 10, and 12 mmHg CO_2_ pneumoperitoneum, respectively.

This research conformed to the institutional protocol for euthanasia (http://risetcenterfk.ulm.ac.id/euthanasia/) and has been approved by the Animal Experimentation Ethical Committee, Research Center, Faculty of Medicine, Lambung Mangkurat University, Banjarmasin, Indonesia (#116-118/KEPK-FK.UNLAM/EC/X/2015).

### Laparoscopic procedure and sample collection

**Fig. 1:**
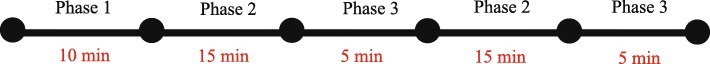
Pneumoperitoneum phase. Desufflation was performed twice during the laparoscopy: the first desufflation in the middle of the laparoscopy due to the change of instruments and the final one was before the end of the surgery.

Laparoscopic surgery was performed under sterile conditions. Abdominal hair was shaved and disinfected with betadine (10% povidone-iodine solution; PT Mahakam Beta Farma, Indonesia No. Reg. DTL 1013705941A1). The rats were anaesthetized with an intramuscular injection of ketamine HCl (KTM-10; PT Guardian Pharmatama, No. Reg. DKL0408013443B1) at a dose of 10 mg/kg BW. Standard CO_2_ and CO_2_ automatic insufflators (Gimmi, Gimmi®GmbH, Germany, 2000) were used for pneumoperitoneum. CO_2_ pneumoperitoneum was divided into three phases for all groups as follows: a) phase 1: stabilization. After the cannula was performed, the rats were stabilized; b) phase 2: pneumoperitoneum (insufflation). CO_2_ insufflators were connected via an 18-gauge needle by peritoneal penetration. The pressure was set at 8, 10, or 12 mmHg; and c) phase 3: desufflation. Desufflation was performed twice during the laparoscopy: the first desufflation was in the middle of the laparoscopy due to the change of instruments, while the final one was before the end of the surgery (Fig. 1).

### Stress oxidative analysis

Twenty-four hours after laparoscopy, the rats were sacrificed in ketamine injections and followed by neck dislocations. Heart blood samples were collected using vacutainers containing ethylenediaminetetraacetic acid. Samples were centrifuged for 10 min at 3000 rpm, and the supernatants were harvested and stored at -20°C until further analysis. The supernatant used for the analysis H_2_O_2_, CAT, SOD, and MDA. Analyses were performed in the chemical/biochemistry laboratory of Universitas Lambung Mangkurat.

#### Hydrogen peroxide (H_2_O_2_)

The level of H_2_O_2_ was measured using the colourimetric modification method [15]. Briefly, 0.01 M phosphate buffer (pH 7.0) was prepared using 5% K_2_Cr_2_O_7_, 150 mL glacial acetate, and 0.2 M H_2_O_2_. Adjust to a standard curve with a concentration of H_2_O_2_ of 20–160 μmol. Serum was added to the phosphate buffer (1:5); 1 mL of this mixture was added to 2 mL glacial acetate. The sample was boiled to remove the blue precipitate and measured by colorimetry at λ = 570 nm. The results are expressed as μM.

#### Catalase (CAT)

The Aebi method was used for measuring CAT activity [16]. Guaiac solution was prepared with phosphate-buffered saline (pH 7), and the serum sample and 10 mM H_2_O_2_ were added (1 mL each). The absorbance was measured at 470 nm (A1) and 570 nm (A2).

#### Superoxide dismutase (SOD)

The Misra and Fridovich method was used to measure SOD activity [17]. Carbonate buffer (100 mM, pH 10.2 using 0.528 g Na_2_CO_3_ in 100 mL sterile water) was prepared. A 500 μL sample was added to 800 μl carbonate buffer and 100 μL 3 mM epinephrine. One milliliter of carbonate buffer was used as the blank solution. The standard solution was 900 μL carbonate buffer + 100 μL 3 mM epinephrine. The absorbance of the sample solution was measured at 480 nm (A0) and repeated after 15 s for A1 then compared to the standard solution. One unit of SOD activity was defined as the amount of enzyme that inhibits the rate of epinephrine oxidation by 50%.

#### Malondialdehyde (MDA)

Buege and Aust's method was used for determining the MDA level by measuring thiobarbituric acid reactive substances [18]. A 0.5 ml sample was prepared, and 1.25 ml 40% TCA, 200 μL 1 N HCl, 0.5 ml sterile water, and 100 μl NaThio were added. The mixtures were heated for 25 min at 100°C, centrifuged for 5 min at 3000 rpm, and the supernatants were harvested. The sample was then diluted with sterile water up to 3 mL. Solutions were measured spectrophotometrically at λ = 500–600 nm.

#### Oxidative stress index (OSI)

OSI is defined as the ratio between MDA and the total activity of SOD and CAT. Therefore, OSI can be calculated using the following equation [19]:

$$ OSI\ \left(\frac{\mu M}{unit}\right)=\frac{MDA}{SOD+ CAT} $$

### Histological analysis of mast cell infiltration and degranulation

Seven days after laparoscopy, the rats were sacrificed in ketamine injections and followed by neck dislocations. The greater omentum, mesenterium, and peritoneum were collected for analysis. The samples were washed in saline, placed in object-glass, and dried at 70°C for 3 min. Samples were stained with toluidine blue for 5 min. Mast cell evaluation was performed using an Olympus (Japan) CX31 microscope and an Olympus U-TV1X-2 lens (100× magnification). Histological images were captured with GraBee version 2.0.0. Mast cell degranulation was measured by dividing the number of degranulated mast cells by the total number of mast cells and expressed as a percentage. Evaluations were performed independently by three pathologists who were blinded to the in 4” (100× magnification).

### Histological analysis of mesothelium thickness

Seven days after laparoscopic, the rats were sacrificed in ketamine injections and followed by neck dislocations. Samples of the liver, small intestine 1 cm from the proximal colon, greater omentum, and peritoneum were collected for analysis. Tissues were fixed in 10% formaldehyde solution and prepared for paraffin blocks. Samples were dehydrated and made using a microtome and stained with hematoxylin and eosin in a thickness of 5 mm. Images were captured with GraBee version 2.0.0, and ImageJ was used to measure the distance from the basal membrane to the edge. Evaluations were performed independently by pathologists who were blinded to the study in 4” (100× magnification).

### Statistical analysis

Data were presented as numbers, percentages, mean ± standard deviation, and median (minimum-maximum). First, we performed using Kolmogorov–Smirnov, and Shapiro–Wilk normality tests and Levene’s homogeneity test. Data transformation methods (power > 1, inverse, log10, and square root) were used for not-normal and not-homogeneous distribution data, followed by one-way analysis of variance, Kruskal–Wallis, or Welch tests for comparison. Multiple range tests used post hoc Tukey’s honestly significant difference test, post hoc Games–Howell, Mann–Whitney, or independent sample tests. A confidence interval of 95% (α=0.05) was used for comparison significance. Spearman correlation with the significance of α=0.01 was used to analyze relationships between variables. The analysis was performed using SPSS version 17.0 and Microsoft Excel 2010.

## Results

### H_2_O_2_ level increased after laparoscopy

**Fig. 2:**
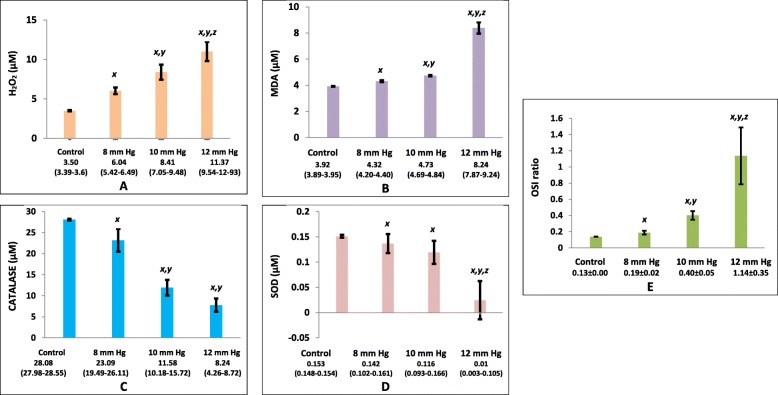
Stress oxidative profiles in serum. Data were presented as mean ± standard deviation or median (minimum-maximum), n=7**. a** H_2_O_2_**, (b)** MDA**, (c)** catalase**,** and **(d)** SOD level were analyzed in Kruskal-Wallis and Mann-Whitney tests**; e** OSI ratio was analyzed using ANOVA and Post-hoc LSD. x*, p*<0.05 (significantly different from the control group). y, *p*<0.05 (significantly different from the P1 [8 mmHg] group). z*, p*<0.05 (significantly different from the P2 [10 mmHg] group).

The H_2_O_2_ level was higher in the intervention groups compared with the control group (3.50 [range, 3.39–3.61] *vs.* 6.04 [range, 5.24–6.49] *vs.* 8.41 [range, 7.05–9.48] *vs.* 11.37 [range, 9.54–12.93] μM for control, P1, P2, and P3 groups, respectively; *p*<0.05) (Fig. 2a).

### MDA level increased after laparoscopy

Lipid membrane peroxidation was analyzed by determining MDA levels 24 h after laparoscopy. Serum MDA was higher in the intervention groups than in the control group (3.92 [range, 3.89–3.95] *vs.* 4.32 [range, 4.20–4.40] *vs.* 4.73 [range, 4.69–4.84] vs. 8.24 [range, 7.87–9.24] μM for control, P1, P2, and P3 groups, respectively; *p*<0.05) (Fig. 2b).

### Antioxidant level of CAT and SOD level decreased after laparoscopy

The CAT level was significantly higher in the control group than in the intervention groups (28.00 [range, 27.98–28.55] *vs.* 23.09 [range, 19.49–26.11] *vs.* 11.58 [range, 10.18–15.72] *vs.* 8.24 [range, 4.26–8.72] μM, for control, P1, P2, and P3 groups, respectively; *p*<0.05) (Fig. 2c).

The SOD level was also significantly higher in the control than in the intervention groups (0.153 [range, 0.148–0.154] *vs.* 0.142 [range, 0.102–0.161] *vs.* 0.116 [range, 0.093–0.166] vs. 0.01 [range, 0.003–0.109] μM for control, P1, P2, and P3 groups, respectively; *p*<0.05) (Fig. 2d).

### OSI increased after laparoscopy

OSI is defined as the ratio between MDA and the total activity of SOD and CAT. Rats that received pneumoperitoneum showed a higher index than in the control group and was directly proportional to the pressure level (0.14 ± 0.00 *vs.* 0.19 ± 0.02 *vs.* 0.40 ± 0.05 *vs.* 1.14 ± 0.35 for control, P1, P2, and P3 groups, respectively, *p*<0.05) **(**Fig. 2e**).**

### Mastocytosis (mast cell infiltration)

**Fig. 3:**
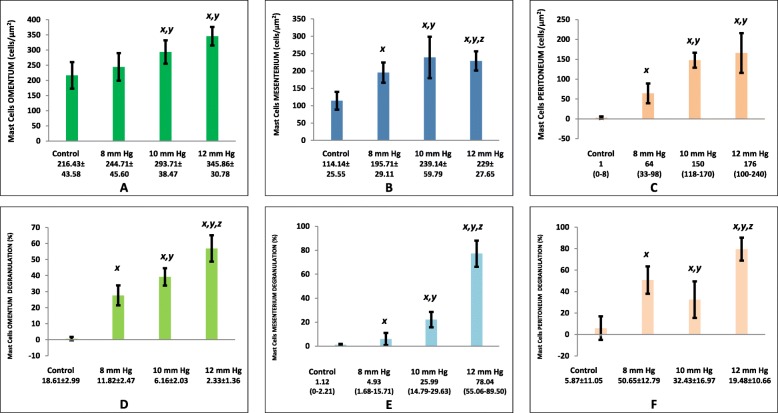
Mast cell infiltration (mastocytosis) and degranulation. Data were presented in mean ± standard deviation or median (minimum-maximum), n=7. Mast cell infiltration in omentum **(a)** and mesenterium **(b)** were analyzed in ANOVA and Post-hoc LSD**.** Mast cell infiltration in peritoneum **(c),** mast cell degranulation in omentum **(d),** and mast cell degranulation in mesenterium **(e)** were analyzed in Kruskal-Wallis and Mann-Whitney**.** Mast cell degranulation in peritoneum **(f)** was analyzed in ANOVA and Post-hoc LSD**.** x*, p*<0.05 (significantly different from the control group). y, *p*<0.05 (significantly different from the P1 [8 mmHg] group). z*, p*<0.05 (significantly different from the P2 [10 mmHg] group).

**Fig. 4:**
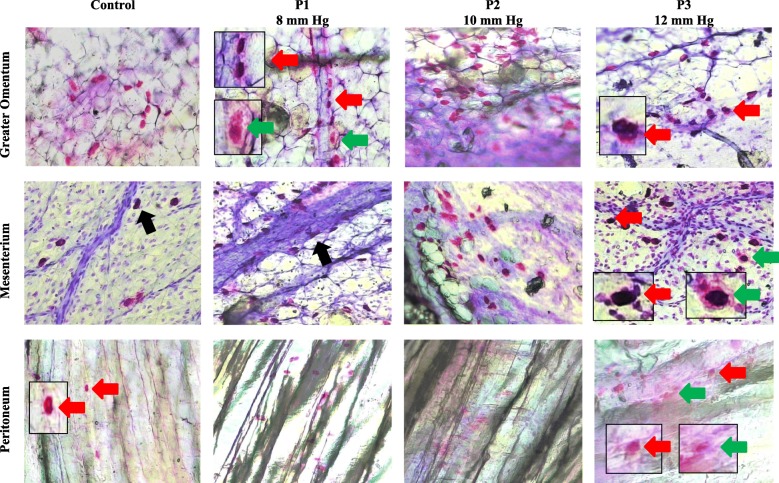
Histological findings of mast cell infiltration (mastocytosis) and degranulation (toluidine blue, 40 X magnifications). Due to increased pressure during laparosocpy, there was an increase in the number and degranulation of mast cells in the omentum, mesenterium, and peritoneum. Red arrows indicate non-degranulated mast cells, and green arrows indicate degranulated mast cells. The mast cells were commonly identified in perivascular (black arrow).

Mastocytosis was significantly higher in the intervention groups than in the control group (216.43 ± 43.58 *vs.* 244.71 ± 45.60 *vs.* 293 ± 38.47 *vs.* 345.86 ± 30.78; 114.14 ± 25.55 *vs.* 195.71 ± 29.11 *vs.* 239.14 ± 59.79 *vs.* 229 ± 27.65; and 1 [range, 0–8] *vs.* 64 [range, 33–98] *vs.* 150 [range, 118–170] *vs.* 176 [range, 100–240] in the greater omentum, mesenterium, and peritoneum, respectively for control, P1, P2, and P3 groups, respectively, *p*<0.05) **(**Fig. 3a-c and 4**).**

### Mast cell degranulation

There was significantly higher mast cell degranulation in the intervention groups than in the control group (0.01 [range, 0–3.21] *vs.* 26.53 [range, 19.3–35.81] *vs.* 40.99 [range, 30.94–46.63] *vs.* 57.23 [range, 42.93–65.23]; 1.12 [range, 0–2.21] *vs.* 4.93 [range, 1.68–15.71] *vs.* 25.99 [range, 14.79–29.63] *vs.* 78.04 [range, 55.06–89.50]; and 0.01 [range, 0–28.6] *vs.* 53.1 [range, 27.27–68.00] *vs.* 24.03 [range, 18.4–66.10] *vs.* 80.85 [range, 65.00–93.50] in the greater omentum, mesenterium, and peritoneum, respectively, for control, P1, P2, and P3 groups, respectively, *p*<0.05) **(**Fig. 3d-f and 4**)**.

### Mesothelium thickness

**Fig. 5:**
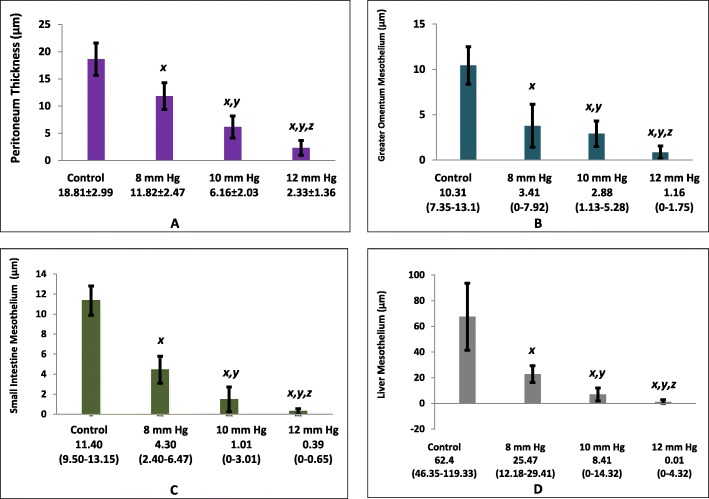
Mesothelium thickness. Mesothelium thickness in peritoneum **(a)** was analyzed in ANOVA and Post-hoc LSD. Mesothelium thickness in greater omentum **(b),** small intestine **(c),** and liver **(d)** were analyzed in Kruskal-Wallis and Mann-Whitney. x*, p*<0.05 (significantly different from the control group). y, *p*<0.05 (significantly different from the P1 [8 mmHg] group). z*, p*<0.05 (significantly different from the P2 [10 mmHg] group).

**Fig. 6:**
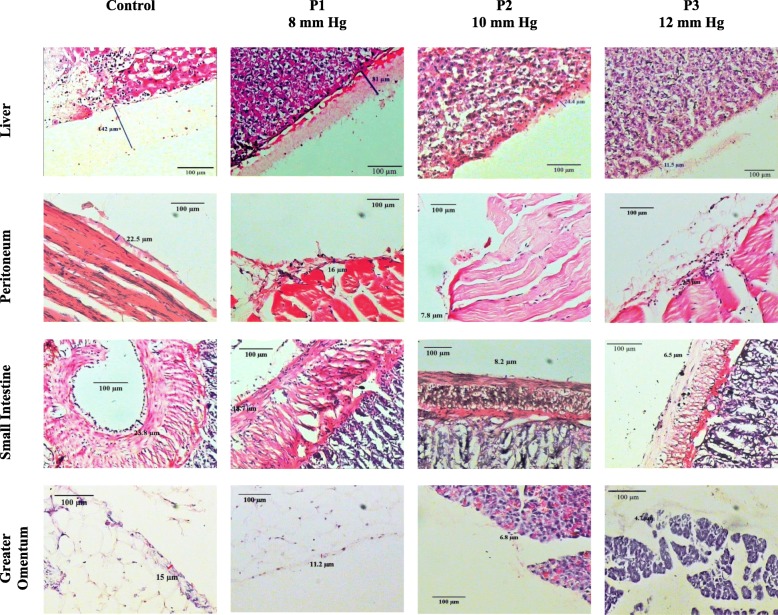
Histological findings of mesothelium thickness. The mesothelium is a layer of squamous cells below the glycocalyx. Due to increased pressure during laparoscopy, there was a decrease in the thickness of the glycocalyx in the liver, peritoneum, small intestine, and greater omentum. (100 × magnification).

The mesothelium was significantly thicker in the control group than in the intervention groups (18.61 ± 2.99 *vs.* 11.82 ± 2.47 *vs.* 6.16 ± 2.03 *vs.* 2.33 ± 1.35; 10.31 [range, 7.35–13.1] *vs.* 3.41 [range, 0–7.92] *vs.* 2.88 [range, 1.13–5.28] *vs.* 1.16 [range, 0–1.75]; 62.4 [range, 46.35–119.33] *vs.* 25.47 [range, 12.18–29.41] *vs.* 8.41 [range, 0–14.32] *vs.* 0.01 [range, 0–4.32]; and 11.40 [range, 9.50–13.15] *vs.* 4.30 [range, 2.40–6.47] *vs.* 1.01 [range, 0–3.01] vs. 0.39 [range, 0–0.65] μM in the peritoneum (Fig. 5a), greater omentum (Fig. 5b), liver (Fig. 5c), and small intestine (Fig. 5d), respectively, for control, P1, P2, and P3 groups, respectively, *p*< 0.05) (Fig. 6).

### Associations between mastocytosis and mast cell degranulation with mesothelium thickness

There was a significant correlation between mastocytosis and mast cell degranulation and change in mesothelium thickness in the peritoneum, greater omentum, small intestine, and liver mesothelium (*p*<0.01).

## Discussion

Laparoscopy has fewer potential complications than laparotomy [20], such as adhesions, intestinal obstruction, infertility, and chronic pelvic pain [2, 9]. CO_2_ insufflation for good visualization during surgery causes hypoxic and splenic ischemia and triggers peritoneal adhesion [9, 21]. The duration of insufflation is proportional to the risk of complications. Insufflation causes mesothelium ischemia and increases hypoxanthine as a product of anaerobic cell metabolism. Cell ischemia produces proteases and activates xanthine oxidase. Both will react with oxygen during desufflation, increasing ROS production [22, 23]. ROS damage the tissue and cause adhesions. Ten percent of the immune cells commonly found in the mesothelium layer are mast cells. Mast cells are part of the innate immune system, increase and attract other immune cells after laparoscopy [23]. Our study showed that laparoscopy induces oxidative stress, increases infiltration and degranulation of mast cells, which causes morphological damage to the mesothelium membrane.

ROS consists of free radicals and not free radicals. Superoxide anions, hydroxyl radicals, and nitric oxide are unstable and are converted to free radicals by SOD to H_2_O_2_. These radicals cause extensive intracellular damage to DNA, proteins, or lipid membranes, induce inflammatory reactions [24], and adhesion formation after surgery [25]. Serum H_2_O_2_ is a reliable parameter for measuring oxidant levels and reflects ischemic reperfusion injury after laparoscopy. Our study found that H_2_O_2_ concentration significantly increased proportionally to insufflation pressure. Increased ROS production by peritoneal macrophages has also been reported as an increase in superoxide anion [26].

Increasing ROS due to the decrease of enzyme activity that converting the oxidants to H_2_O_2_, subsequently to water and oxygen. SOD is the responsible enzyme for the H_2_O_2_ formation. CAT and glutathione peroxidase convert H_2_O_2_ into water and oxygen. Our study found that SOD and CAT levels decreased significantly in the intervention groups. Oksuz *et al.* showed that SOD levels decrease after laparoscopy and antioxidant injections increase SOD levels [27]

Oxidative stress can be assessed through damage to the lipid membrane, DNA, and protein degradation. Lipid membrane peroxidation was analyzed through MDA levels 24 hours after laparoscopy. Our study found that MDA levels increase significantly in line with pressure. Langendonckt *et al.* found that human plasma MDA levels increase 5 minutes after laparoscopy. There was no significant increase in MDA 24 hours postoperatively [25]. MDA is the final product of oxidative stress, and our data support that desufflation and reperfusion contribute to the formation of ROS.

OSI is the ratio between cell damage (MDA) and the availability of endogenous antioxidants (the amount of SOD and CAT). Our study found that OSI increased significantly according to increased insufflation pressure and compatible with the previous report [21].

Mastocytosis is an abnormal mast cell proliferation [28], caused by mutations of CD117 [29]. Mastocytosis can be analyzed by histological examination [30]. Mast cells have a unique characteristic, released from bone marrow as immature progenitor mast cells into circulation while expressing various surface molecules and integrins precisely according to their home tissues [31]. In peripheral tissues, mast cells will mature, producing mediators according to their microenvironment. Once activated, mast cells can respond accurately to cell damage [32, 33].

Our study revealed that a significant increase in mast cell infiltration 7 days after laparoscopy, and the increase was proportional to the level of pressure. Mastocytosis can increase in peripheral tissue or additional recruitment from bone marrow [34]. Sonoda *et al.* found that skin generates mature mast cells [35]. Migration of mast cell progenitors to the peritoneal membrane depends on the integrins αMβ2 and αIIbβ3 [36, 37]. Expression of these integrins is highly specific; it differs from VCAM-1 and the integrins α4β1 and α4β4, which are needed for intestinal migration [38]. Those findings are consistent with the study of Jamur, who demonstrated that mast cell precursors increase in the bone marrow, producing mast cell progenitors that migrate and infiltrate the peritoneal membrane after being depleted with saline [39]. In allergic reactions, tissue mast cell progenitors increase in number following sensitization [40]. Following peritoneal insufflation, Volz *et al.* observed local acidosis in the mesothelium layer, together with endotoxin, nonspecific noxae become a chemotactic factor in attracting immune cell infiltration to the site, as an initial reaction in peritoneal healing [41].

In the resting phase, mast cells develop granules that consist of active metabolites such as biogenic amines, cytokines, and peptides, as well as proteases (tryptase and chymase), which are released upon activation (degranulation). Connective tissue mast cells are known to have 10-fold lower expression of FcƐRI than mucosal mast cells for activation; mast cells can also be activated by non-immune mediators such as complement, substance 48/80, and substance P [42]. Our study found that there was a relationship between mast cell degranulation and mesothelial thickness. The mast cell degranulation increased significantly with increased pressure. These data are supported by Steiner *et al*. Mast cells can also be activated by substance 48/80 and degranulation. Soluble granules become chemokines that attract other immune cells and induce inflammation [43]. FcƐRI can mediate degranulation by producing intracellular ROS through Lyn and Syk activation, which increases the cytoplasmic concentration of calcium [44].

Insufflation creates shear stress and increases intraabdominal pressure. Both are mechanical pathways to stimulate degranulation of local mast cells [45, 46]. Stretching and changes in pressure activate TPRV4 channels of mechanosensitive calcium ions in mast cells, cause calcium to enter the cytoplasm. Calcium has a vital role in releasing stored proteases because increased intracellular calcium stimulates and activates mast cells [47].

Our study also presented that high pressure caused a significant decrease in mesothelium thickness, which was measured in the parietal peritoneum, small intestine, liver, and greater omentum. The damage might be induced by ischemia-reperfusion injury during insufflation and desufflation, which is known to increase ROS and nitrogen species harmful to parenchyma and vascular [48, 49]. The glycocalyx is one structure susceptible to oxidative damage [50]. The results are supported by the study of Debray-Gracia *et al.,* in which oxidative stress was induced in the diabetic peritoneum by peritoneal dialysis [51]. It was found that protein carbonylation, an oxidative damage marker, increased, tight junctions were altered, and the thickness of the sub-mesothelium and connective tissue decreased. Ten minutes after oxidant exposure, mesothelial cells begin to die through necrosis and apoptosis, resulting in a reduction of the cell population, and 30 days later, the mesothelium layer began to repopulate together with the development of fibrotic tissue [52], an early stage of adhesion formation. Oxidants, such as extracellular H_2_O_2_, can induce intracellular ROS by activating PKC in a human peritoneum mesothelial cell (*in vitro* study) [53].

Mesothelium thinning was significantly correlated with mastocytosis and mast cell degranulation. Mast cells exocytosis, released protease tryptase and chymase, directly or indirectly decreased ECM, fibronectin, and collagen of the mesothelium [54]. ZO-1 tight junction proteins altered and changed cell permeability [55–58]. Overall, mast cell degranulation triggered mesothelium thinning, caused oxidant-induced glycocalyx damage, and led to the development of fibrotic and adhesion formation.

Our study proved that mast cells caused mesothelium damage. Degranulation of mast cells after laparoscopic induced mesothelial cell damage and led to complications, i.e., intra-abdominal adhesion.

There was a limitation in our study that used systemic blood samples oxidative parameters and did not determine the direct correlation between oxidative stress and mast cells in the abdominal cavity. Three insufflation pressures significantly caused the expected damage. Future study, in particular, about pressures lower than 8 mmHg, additional variables to explain the effects of oxidative stress (i.e., using peritoneal fluid samples) and their implications for mast cells and products (i.e., histamine, tryptase, chymase), as well as treatment to avoid complications, needs to be done. A deeper understanding of mast cells in the mesothelium layer can be expected to improve laparoscopy in the future.

## Conclus**i**ons

Our study shows that laparoscopy in rats causes stress oxidative, results in a decrease in mesothelial thickness that is correlated with mast cell infiltration and degranulation. Degranulation of mast cells results in mesothelial cell damage and makes the loss of glycocalyx protective function and might cause complications, such as an intra-abdominal adhesion.

## Data Availability

The datasets used and/or analyzed during the current study are available from the corresponding author on reasonable request.

## Abbreviations

CO_2_: Carbon dioxide
CAT: Catalase
ECM: Extracellular matrix
H_2_O_2_: Hydrogen peroxide
MDA: Malondialdehyde
OSI: Oxidative stress index
PAR-2: Proteinase-activated receptor-2
ROS: Reactive oxygen species
SOD: Superoxide dismutase.
